# Management strategy of primary ovarian mucinous carcinoid tumor: A rare case report

**DOI:** 10.1097/MD.0000000000039772

**Published:** 2024-09-20

**Authors:** Byung Su Kwon, Da Young Kang, Kiyong Na, Youngsun Kim

**Affiliations:** a Department of Obstetrics and Gynecology, Kyung Hee University College of Medicine, Kyung Hee University Medical Center, Seoul, Korea; b Department of Pathology, Kyung Hee University College of Medicine, Kyung Hee University Medical Center, Seoul, Korea.

**Keywords:** case report, primary ovarian mucinous carcinoid tumor

## Abstract

**Rationale:**

Primary ovarian carcinoid tumors are rare neoplasms, first reported in 1939, with approximately 30 cases reported thus far. It is categorized into insular, trabecular, strumal, and mucinous types. Mucinous forms are extremely rare, comprising < 2% of all primary ovarian carcinoid tumors.

**Patient concerns:**

A 40-year-old gravida 3, para 0 woman visited our clinic with a 3-month history of lower abdominal pain. Ultrasound and abdominal pelvic computed tomography revealed a large, poorly enhancing soft tissue mass in the right adnexa (about 9.4 × 7.0 × 6.8 cm sized). Laparoscopic surgery was performed to a definitive diagnosis, including right salpingo-oophorectomy, left ovarian biopsy, and ascites washing cytology.

**Diagnosis:**

The patient was diagnosed with primary ovarian mucinous carcinoid tumor and received related treatment.

**Outcomes:**

After treatment, the patient symptoms improved, and he was discharged.

**Lessons:**

Approximately 40% of primary ovarian carcinoid tumors with insular morphology present in pure form, and mucinous forms are extremely rare. At present, the main diagnostic methods in cases of primary ovarian mucinous carcinoid tumor include macroscopic examination, histopathology and imaging examination. The main treatment modalities for primary ovarian mucinous carcinoid tumor are surgery. postoperative chemotherapy remains controversial.

## 1. Introduction

Ovarian carcinoid tumors are well-differentiated neuroendocrine tumors (NETs) that account for 0.1% of all ovarian neoplasms.^[1–3]^ NETs are a heterogeneous group of neoplasms most commonly occurring in the gastrointestinal tract or the lungs and more frequent are gastrointestinal tumors.^[2]^ NETs in the gynecologic tract are uncommon and account for about 2% of all gynecologic malignancies but may also be metastatic from other sites.^[2]^ Approximately 30% to 40% of patients with well-differentiated NETs present with carcinoid syndrome (CS).^[3]^ CS is a paraneoplastic syndrome associated with the secretion of several humoral factors, such as polypeptides, vasoactive amines, and prostaglandins.^[3]^ The main symptoms of CS are episodic facial flushing that may be accompanied by hypotension and tachycardia, diarrhea, bronchoconstriction, venous telangiectasia, dyspnea and ultimately fibrotic complications such as mesenteric and retroperitoneal fibroses and carcinoid heart disease.^[4]^

Primary ovarian carcinoid tumors are rare neoplasms, first reported in 1939, with approximately 30 cases reported thus far.^[5]^ It is categorized into insular, trabecular, strumal, and mucinous types.^[6]^ Approximately 40% of primary ovarian carcinoid tumors with insular morphology present in pure form, and mucinous forms are extremely rare, comprising < 2% of all primary ovarian carcinoid tumors.^[7,8]^

Their age of presentation ranges from 14 to 74 years, with a mean age of 43 years.^[9]^ Most tumors present as enlarged masses or abdominal distension and sometimes present abnormal uterine bleeding.^[9]^

## 2. Case report

**Figure 1.**
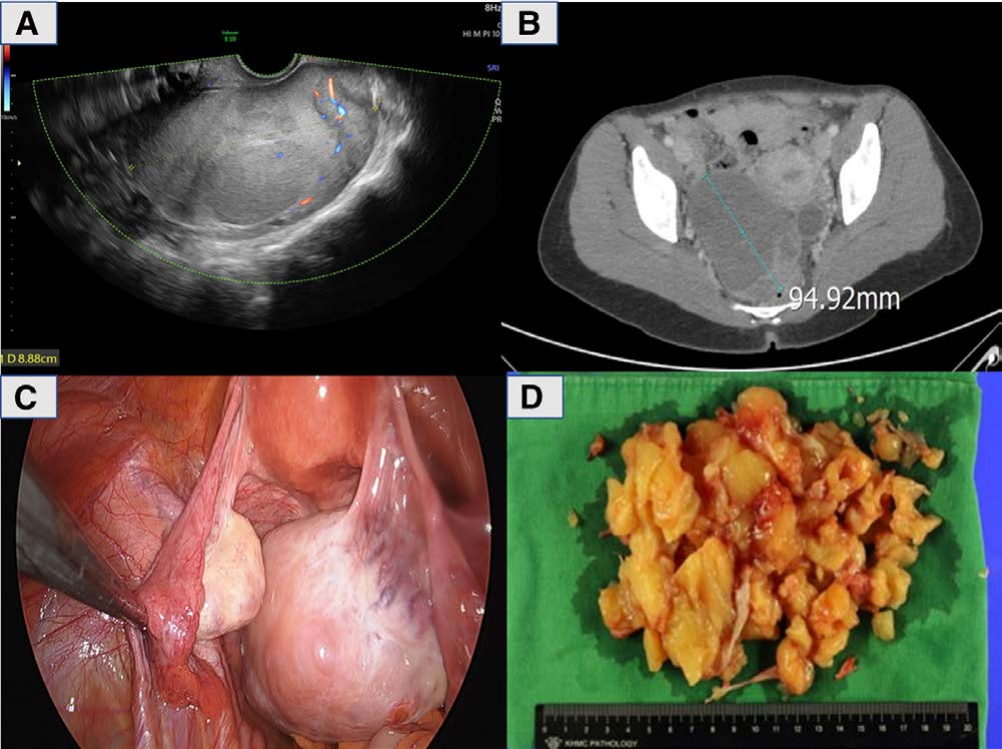
Findings of right ovrian mass. (A) Ultrasound showed about 9cm right ovarian tumor. (B) Abdominal computed tomography (CT) revealed a large poorly enhancing soft tissue mass at the right adnexa (about 9.4 × 7.0 × 6.8 cm sized). AJCC. (C) Laparoscopic surgery finding. (D) Gross examination of the tumor was performed in the fragmented tissue state, measuring 15 cm in aggregation. The tumor was yellow-tan, soft mass and showed abundant myxoid area.

A 40-year-old gravida 3, para 0 woman visited our clinic with a 3-month history of lower abdominal pain. Her medical and family histories were unremarkable. Cancer antigen 125 and carcinoembryonic antigen levels were within normal limits. Gynecological pelvic examination, ultrasound and abdominal pelvic computed tomography (CT) revealed a large, poorly enhancing soft tissue mass in the right adnexa (about 9.4 × 7.0 × 6.8 cm sized) (Fig. 1A and B). Positron emission tomography-CT revealed no residual hypermetabolic lesions in the pelvic cavity. She showed no CS.

A tumor was confirmed in the right ovary during surgery through laparoscopy. Macroscopically, the contralateral ovary showed no abnormalities (Fig. 1C). The patient underwent fertility-sparing staging surgery, including right salpingo-oophorectomy, left ovarian biopsy, and ascites washing cytology (Fig. 1C).

The gross examination of the tumor was performed on a fragmented tissue, measuring 15 cm. The tumor presented as a soft yellow-tan mass with an abundant myxoid area (Fig. 1D), displaying no cystic changes or necrosis.

**Figure 2.**
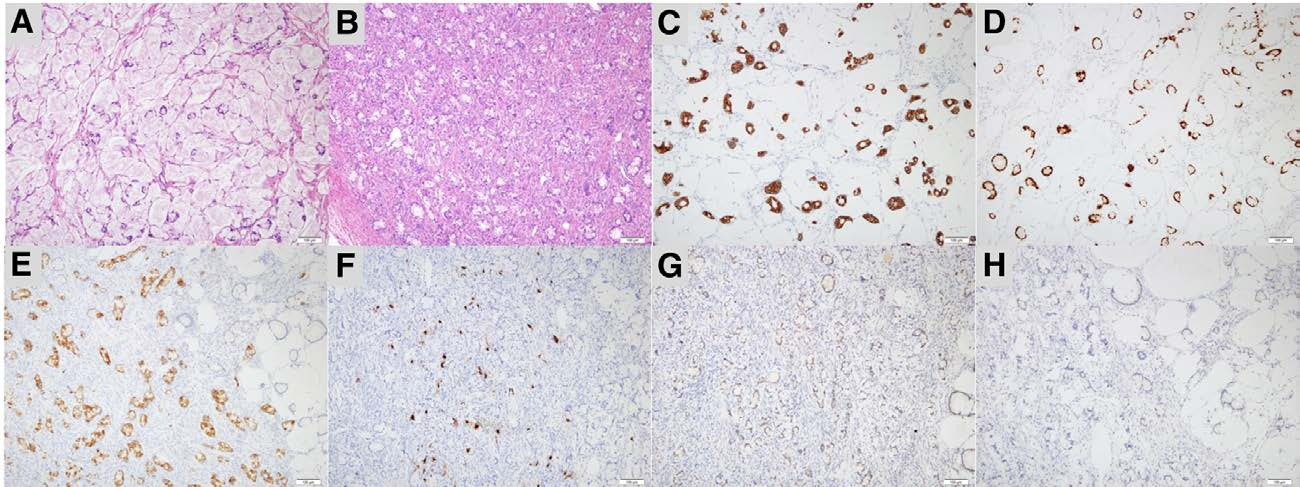
Histopathologic findings. (A) Most of the tumor (approximately 90%) in the slides examined was composed of evenly spaced discrete mucinous glands floating within pools of mucin. (B) Focal nodular lesion occupying small proportion of the tumor (approximately 10%) showed more cellular areas, which exhibited well-formed mucinous glands, and clustered or singly scattered signet-ring cells embedded in fibromatous stroma. (C–H) Immunostaining.

Approximately 90% of the tumor on the examined slides was composed of evenly spaced discrete mucinous glands floating within mucin pools (Fig. 2A). Thin fibrous septa containing a few fibroblasts and capillary-sized blood vessels divided the mucin pools, simulating the alveolar structure of the lungs. The glands had small central lumens, which were empty or contained pale eosinophilic material. The lumens were lined with goblet and columnar cells with a pale eosinophilic cytoplasm. Focal nodular lesions occupying a small proportion of the tumor (approximately 10%) exhibited more cellular areas, along with well-formed mucinous glands and clustered or singly scattered signet-ring cells embedded in the fibromatous stroma (Fig. 2B). Overall, cytological atypia was minimal and mitotic counts ranged from 0 to 1 per 10 high-power fields. The remaining ovarian tissue did not exhibit any abnormal characteristics, except for a small follicular cyst. Immunostaining of the tumor cells revealed diffusely strong positivity for CDX2 (Fig. 2C) and CK20 (Fig. 2D); focally strong positivity for synaptophysin (Fig. 2E) and chromogranin A (Fig. 2F); patchy weak positive for PAX-2 (Fig. 2G); and negative for NSE, PAX-8 (Fig. 2H), CD56, and CK7. The Ki-67 labeling index was approximately 1%. Spindle cells in the fibromatous stroma were focally positive for SMA but negative for desmin and S100. After tumor tissue microdissection, Oncomine Comprehensive Assay Plus (Thermo Fisher Scientific, MA)-based targeted next-generation sequencing was performed. The tumors harbored a 19p gain but without sequence change mutations, translocations, or copy number alterations, suggesting amplification or deletion.

Histological examination confirmed primary ovarian mucinous carcinoid tumors (POMCT).

The patient was not treated other chemotherapy and discharged 3 days after surgery. After 9 months of follow-up in an outpatient clinic, there was no recurrence.

## 3. Discussion

POMCT are treated by surgical excision. According to the literature, patients with disease confined to 1 ovary show an excellent survival. However, if the disease is in the advanced stage, the outcome in these patients is poor. Therefore, a staging operation is recommended for treating POMCT. However, the optimal extent of surgery has not yet been established. Diagnosing POMCT using imaging tests before treatment poses challenges in determining the extent of surgery. According to the literature review, in some cases, only unilateral salpingo-oophorectomy was performed, whereas in other cases, the tumors were bilaterally removed. Furthermore, in some cases, a hysterectomy was performed. In our cases where the patient wishes to preserve fertility, a reasonable approach involves sparing the uterus and contralateral ovary, assuming that there are no metastatic findings on preoperative imaging.

Epithelial ovarian cancer is surgically staged according to the 2017 8th Edition American Joint Committee on Cancer (AJCC) and the International Federation of Gynecology and Obstetrics (FIGO) Tumor, Node, Metastasis (TNM) classification system (Table 1).^[10]^ Although it has not yet been established, it would be good to use this staging system to diagnose ovarian carcinoid tumor.

**Table 1:** Ovarian carcinoma FIGO staging (AJCC 8th edition).

FIGO stage				Criteria
I	A			Tumor limited to 1 ovary (capsule intact) or fallopian tube, no tumor on ovarian or fallopian tube surface; no malignant cells in ascites or peritoneal washings
	B			Tumor limited to both ovaries (capsules intact) or fallopian tubes; no tumor on ovarian or fallopian tube surface; no malignant cells in ascites or peritoneal washings
	C	1		Surgical spill
		2		Capsule ruptured before surgery or tumor on ovarian or fallopian tube surface
		3		Malignant cells in ascites or peritoneal washings
II	A			Extension and/or implants on the uterus and/or fallopian tube(s) and/or ovaries
	B			Extension to and/or implants on other pelvic tissues
III	A	1	¡	Metastasis up to and including 10 mm in greatest dimension
			¡¡	Metastasis more than 10 mm in greatest dimension
		2		Microscopic extrapelvic (above the pelvic brim) peritoneal involvement with or without positive retroperitoneal lymph nodes
	B			Macroscopic peritoneal metastasis beyond pelvis 2 cm or less in greatest dimension with or without metastasis to the retroperitoneal lymph nodes
	C			Macroscopic peritoneal metastasis beyond the pelvis more than 2 cm in greatest dimension with or without metastasis to the retroperitoneal lymph nodes (includes extension of tumor to capsule of liver and spleen without parenchymal involvement of either organ)
IV	A			Pleural effusion with positive cytology
	B			Liver or splenic parenchymal metastases; metastases to extra-abdominal organs (including inguinal lymph nodes and lymph nodes outside the abdominal cavity); transmural involvement of intestine

Owing to the paucity of data available on POMCT, the efficacy of adjuvant chemotherapy remains controversial. In addition, a unanimous consensus regarding the choice of drugs for treatment is lacking. Further studies are required to determine the appropriate chemotherapy regimen.

Only a few cases of POMCT have been documented till date, and most of the patients with stage I disease remained disease-free after the operation for up to 10 years.^[6]^ But some cases demonstrated a more aggressive clinical course compared to that reported in previous literature.^[6]^ Ovarian carcinoid tumors are relatively chemoresistant compared with epithelial ovarian cancers.^[6]^

## 4. Conclusions and lessons learned from this case

Primary ovarian carcinoid tumors are uncommon neoplasms that typically arise in the context of a mature cystic teratoma. This entity is divided into insular, trabecular, mucinous, and strumal subtypes based on the predominant architectural pattern and presence or absence of mucinous epithelium and thyroid tissue.

Among them, mucinous type of ovarian carcinoid tumor is extremely rare. Most of the patients presented with symptoms of abdominal distension and some presented with abnormal uterine bleeding. Some patients show symptoms of CS. In principle, surgical treatment is performed after diagnosis through imaging tests. It is recommended that surgical excision is performed in accordance with ovarian cancer

staging, and there is no established chemotherapic effects after surgery. More research is needed to determine the appropriate scope of surgery, treatment method, and prognosis.

## Author contributions

**Conceptualization:** Youngsun Kim, Byung Su Kwon, Da young Kang, Kiyong Na.

**Investigation:** Byung Su Kwon, Da young Kang.

**Methodology:** Youngsun Kim, Byung Su Kwon, Da young Kang, Kiyong Na.

**Resources:** Byung Su Kwon, Da young Kang.

**Supervision:** Youngsun Kim.

**Writing – original draft:** Byung Su Kwon, Da young Kang.

**Writing – review & editing:** Youngsun Kim, Kiyong Na.
